# The impact of dual energy CT imaging on dose calculations for pre-clinical studies

**DOI:** 10.1186/s13014-017-0922-9

**Published:** 2017-11-21

**Authors:** Ana Vaniqui, Lotte E. J. R. Schyns, Isabel P. Almeida, Brent van der Heyden, Stefan J. van Hoof, Frank Verhaegen

**Affiliations:** Department of Radiation Oncology (MAASTRO), GROW – School for Oncology and Developmental Biology, Maastricht University Medical Center, Maastricht, the Netherlands

**Keywords:** Dual energy CT, Small animal radiotherapy, Dose calculations, Tissue segmentation, Monte Carlo, Pre-clinical

## Abstract

**Background:**

To investigate the feasibility of using dual-energy CT (DECT) for tissue segmentation and kilovolt (kV) dose calculations in pre-clinical studies and assess potential dose calculation accuracy gain.

**Methods:**

Two phantoms and an ex-vivo mouse were scanned in a small animal irradiator with two distinct energies. Tissue segmentation was performed with the single-energy CT (SECT) and DECT methods. A number of different material maps was used. Dose calculations were performed to verify the impact of segmentations on the dose accuracy.

**Results:**

DECT showed better overall results in comparison to SECT. Higher number of DECT segmentation media resulted in smaller dose differences in comparison to the reference. Increasing the number of materials in the SECT method yielded more instability. Both modalities showed a limit to which adding more materials with similar characteristics ceased providing better segmentation results, and resulted in more noise in the material maps and the dose distributions. The effect was aggravated with a decrease in beam energy. For the ex-vivo specimen, the choice of only one high dense bone for the SECT method resulted in large volumes of tissue receiving high doses. For the DECT method, the choice of more than one kind of bone resulted in lower dose values for the different tissues occupying the same volume. For the organs at risk surrounded by bone, the doses were lower when using the SECT method in comparison to DECT, due to the high absorption of the bone. SECT material segmentation may lead to an underestimation of the dose to OAR in the proximity of bone.

**Conclusions:**

The DECT method enabled the selection of a higher number of materials thereby increasing the accuracy in dose calculations. In phantom studies, SECT performed best with three materials and DECT with seven for the phantom case. For irradiations in preclinical studies with kV photon energies, the use of DECT segmentation combined with the choice of a low-density bone is recommended.

## Background

Pre-clinical radiation studies with small animal models play a significant role in the understanding of cancer radiobiology. Such studies also aim towards mimicking human treatment capabilities so that specific validated radiation therapies in animal models can be successfully translated into patient radiotherapy (RT) trials [1]. Accurate pre-clinical radiation targeting requires accurate image guiding. For the various stages of target delineation, treatment planning, dose calculation, beam delivery, and subsequent outcome assessments, precise identification of different tissues and structures is of paramount importance.

Computed Tomography (CT) is the most frequently used imaging modality for RT [2]. Commercial pre-clinical irradiators are equipped with an x-ray tube, which besides the irradiation, is used to acquire high-resolution cone beam CT (CBCT) images (about 100–200 μm) [3].

Small animal irradiation is preferably performed with kilovolt (kV) photons [4], in contrast to human radiotherapy which is mostly performed with megavolt (MV) photons. In the kV energy range, the photo-electric effect is increasingly important and its interaction probability is strongly dependent on the effective atomic number of the tissues (*Z*_*eff*_^3 ~4^) [3]. In current practice, quantitative information on tissues is mostly obtained by single energy CT (SECT) in the form of attenuation coefficients (or CT numbers, expressed by Hounsfield Units, HU). In Monte Carlo (MC) dose calculations, every voxel of the CT scan has a mass density assigned based on the HU value through an empirical calibration.

Tissue identification based on SECT has been shown to lead to errors in dose calculations in the kV-MV energy range [5] and due to the strong dependence of the photoelectric cross sections on the atomic number of the tissues, such errors are amplified in the low-energy photon range [6]. In addition, dose calculation algorithms for kV irradiations of small animals need supplementary information to voxel densities, such as tissue type – as it cannot be assumed the medium is water in kV irradiations. This information can be provided from either SECT or dual energy CT (DECT) images.

The aim of this study is to investigate the feasibility of using dual-energy CBCT for tissue segmentation and kV dose calculations in pre-clinical studies. The main objectives are to assess potential dose calculation accuracy gain from DECT and to establish imaging protocols that allow accurate dose calculations.

While this work has no direct clinical implications, its underlying aim is to perform dose calculations as accurately as possible so as to enable rigorous subsequent clinical translation.

## Methods

### Micro irradiator

The X-RAD 225Cx (Precision X-Ray, North Branford (CT), United States) [4, 7] micro irradiator consists of a dual-focus X-ray tube with a maximum tube potential of 225 kV (225 Cx, Comet, Switzerland) and a 20^o^ angled tungsten stationary target. The X-ray tube acts as photon source for imaging using the small focal spot, and treatment using the large focal spot. Photons are filtered through a 0.8 mm beryllium exit window and additional 2.0 mm filter cassette made of aluminium for imaging or 0.32 mm filter cassette made of copper [8] for irradiation purposes. The source to isocentre distance was fixed at 303.6 mm.

### Extracting information from SECT and DECT methods

**Fig. 1:**
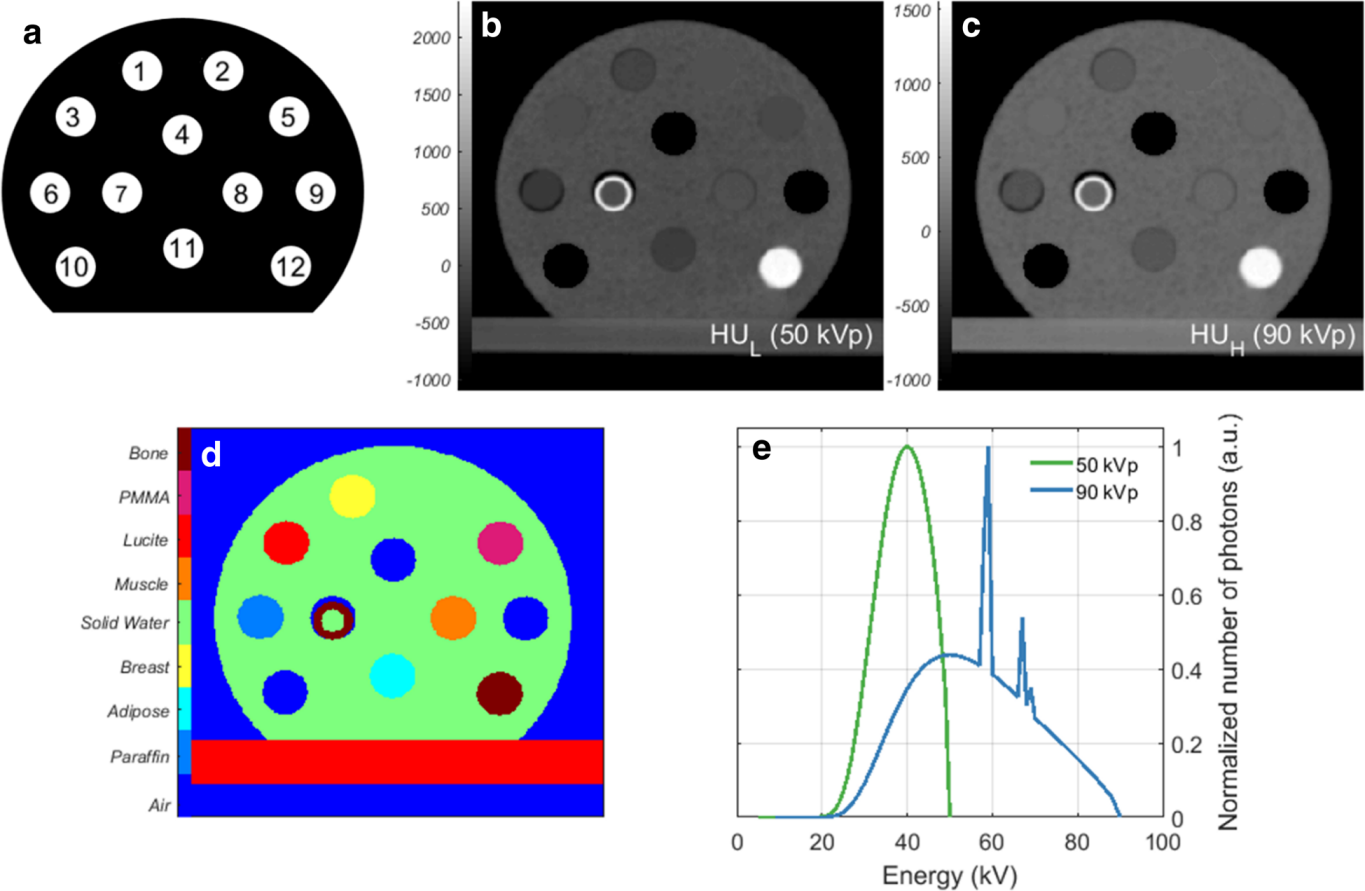
**a** Phantoms are made of Solid Water and contain twelve inserts of tissue-equivalent materials, one set of materials for the calibration phantom and one set of materials for the validation phantom. **b** Central slice of the CT scan at 50 kVp and (**c)** 90 kVp. **d** Reference material map. **e** 50 kVp and 90 kVp photon spectra used for SECT and DECT

For this study, two geometrically identical cylindrical mini-phantoms (SmART Scientific Solutions BV, Maastricht, the Netherlands) of 3 cm diameter and 1 cm length were scanned (Fig. 1a). They are composed of a Solid Water bulk and twelve cylindrical inserts of 3.5 mm diameter and 1 cm length. The composition of the inserts, the relative electron density (*ρ*_*e*_)^1^ and the effective atomic number (*Z*_*eff*_)^2^ provided by the manufacturer are listed in Table 1. The phantom cross-section is consistent with the overall size of the mouse, both head and pelvis, further used in this study.

**Table 1:** Reference values of mass density (*ρ*), relative electron density (*ρ*_*e*_), effective atomic number (*Z*_*eff*_) and elemental composition of the tissue-substitute materials present in the calibration and validation mini-phantoms

	Material	[g/cm^3^]			Mass percentage (%)				
n°		*ρ*	*ρ*_*e*_	*Z*_*eff*_	H	C	N	O	Z > 8
Calibration Phantom									
1	AP6	0.947	0.928	6.210	9.06	72.30	2.25	16.27	F(0.13)
2	Solid Water	1.022	0.992	7.735	8.00	67.30	2.39	19.87	Cl(0.14), Ca(2.31)
3	IB3	1.134	1.086	10.418	6.67	55.64	1.96	23.52	P(3.23), Cl(0.11), Ca(8.86)
4	SR2	1.051	1.047	6.090	10.83	72.54	1.69	14.86	Cl(0.08)
5	CB2–30%	1.331	1.276	10.898	6.68	53.48	2.12	25.61	Cl(0.11), Ca(12.01)
6	BR12	0.980	0.956	6.931	8.59	70.11	2.33	17.90	Cl(0.13), Ca(0.95)
7	Air	0.001	0.001	7.714			75.47	23.20	Ar(1.28)
8	Water	1.000	1.000	7.477	11.20			88.80	
9	B200	1.152	1.103	10.423	6.65	55.52	1.98	23.64	P(3.24), Cl(0.11), Ca(8.87)
10	LV1	1.096	1.064	7.736	8.06	67.01	2.47	20.01	Cl(0.14), Ca(2.31)
11	SB3	1.822	1.695	13.638	3.41	31.41	1.84	36.50	Cl(0.04), Ca(26.81)
12	CB2–50%	1.559	1.469	12.538	4.77	41.63	1.52	32.00	Cl(0.08), Ca(20.02)
Validation Phantom									
1	BR12	0.980	0.956	6.931	8.59	70.11	2.33	17.90	Cl (0.13), Ca (0.95)
2	Teflon	2.153	1.860	8.461		24.00			F (76)
3	Lucite	1.180	1.146	6.529	8.05	59.98		31.96	
4	Air	0.001	0.001	7.714			75.47	23.20	Ar (1.28)
5	PMMA	1.190	1.156	6.529	8.05	59.98		31.96	
6	Paraffin	0.930	0.959	5.483	14.90	85.10			
7	Water	1.000	1.000	7.477	11.20			88.80	
8	Muscle	1.062	1.041	7.588	9.10	69.70	2.10	16.80	Cl (0.10), Ca (2.20)
9	Air	0.001	0.001	7.714			75.47	23.20	Ar (1.28)
10	Air	0.001	0.001	7.714			75.47	23.20	Ar (1.28)
11	Adipose	0.967	0.956	6.439	10.00	71.30	1.80	16.40	Cl (0.20), Ca (0.30)
12	Bone	1.600	1.507	11.895	4.83	37.03	0.97	35.66	Mg (6.19), Cl (0.05), Ca (15.24)

The mini phantoms were imaged using the CBCT imager (resolution 1024 × 1024 pixels) integrated in the small animal irradiator. The images were acquired using a 2.0 mm filter of aluminium for the tube voltages of 50 kVp (low energy) and 90 kVp (high energy) with corresponding currents of 5.59 and 2.08 mA (Fig 1e) shows both photon spectra). The exposures used were of 670.8 mAs and 249.6 mAs yielding the dose of 30 cGy for each energy. The absorbed dose to water at the phantom surface was verified using a TN30012 Farmer ionization chamber (PTW, Freiburg, Germany) according to the AAPM TG-61 protocol for 40–300 kV x-ray beam dosimetry dosimetry (in-air calibration method) [9]. The images were reconstructed using a Feldkamp-Davis-Kress (FDK) backprojection algorithm [10], in a matrix of 341x324x96 with 103.4 × 103.4 × 103.4 μm^3^ voxel size. The acquisition time difference between the two images was of 7 min.

### SECT method

**Fig. 2:**
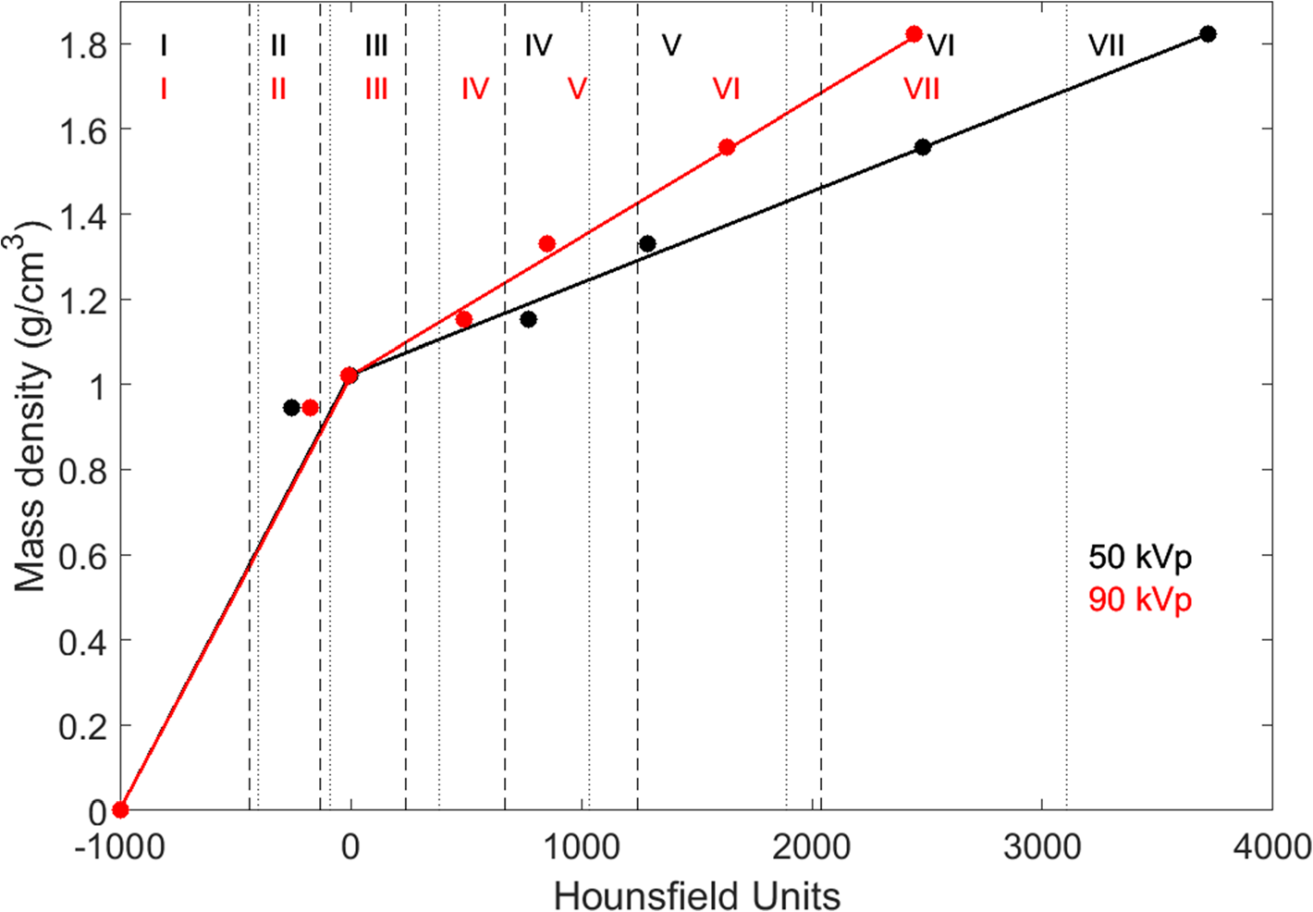
SECT (HU-*ρ*) curve for the calibration phantom at 50 kVp in black and at 90 kVp in red. The vertical dotted (50 kVp) and dashed lines (90 kVp) represent the selected boundaries between media in a segmentation scheme with seven materials (I to VII). The roman numerals I-VII indicate the materials: air, AP6, Solid Water, B200, CB2–30%, CB2–50% and SB3. Other segmentation schemes with a different number of intervals are possible. The dots represent the mean HU value of each material

**Fig. 3:**
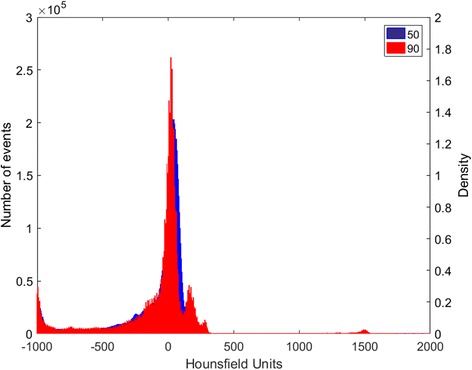
Mass density histogram for 50 and 90 kVp

In the SECT approach, a relationship between HU and mass density (*ρ*) was generated in the form of a (HU-*ρ*) calibration curve. HU are defined as HU = 1000(*μ*/*μ*_w_ − 1), where *μ* and *μ*_*w*_ are respectively the linear attenuation coefficients of the scanned medium and water. Relative electron density ρ_e_ can be converted into mass density ρ through a linear relationship. A piecewise bi-linear HU-*ρ* relationship was generated using the mean HU values of the selected materials in the calibration phantom (Fig. 2). The material segmentation is indicated with vertical lines according to the selected HU ranges. Figure 3 shows the histogram of Hounsfield Units. From the (HU-*ρ*) calibration curve, a density map of the phantom was created. A density to material curve was derived from the density map, which generated the material map. The curve material thresholds were set based on visual inspection of the CT scan as well as on the knowledge of the maximum and minimum HU of each material. In this example, seven materials were chosen for the segmentation. Table 2 shows the mean HU values for each material. A density map was then generated and, according to the chosen segmentation intervals, a material map was generated.

**Table 2:** Mean HU ± standard deviation (σ) per insert for the calibration and validation phantoms for 50 and 90 kVp

Calibration phantom				Validation phantom			
Material	Mean HU ± σ		Insert	Material	Mean HU ± σ		Insert
	50 kVp	90 kVp	N°		50 kVp	90 kVp	N°
Air	−1000 ± 19	−1000 ± 13	7	Air	−1000 ± 17	−1000 ± 13	4, 9, 10
AP6	−248 ± 17	−173 ± 16	1	Paraffin	−281 ± 13	−178 ± 11	6
BR12	−171 ± 19	−115 ± 16	6	Adipose	−226 ± 16	−147 ± 13	11
SR2	−147 ± 18	−64 ± 15	4	BR12	−146 ± 16	−106 ± 13	1
Solid Water	−2 ± 22	−7 ± 20	2	PMMA	−73 ± 12	10 ± 11	5
Water	0 ± 22	0 ± 17	8	Water	−36 ± 15	−15 ± 12	7
LV1	69 ± 23	68 ± 20	10	Lucite	−31 ± 13	46 ± 12	3
IB3	729 ± 54	456 ± 38	3	Muscle	2 ± 14	25 ± 12	8
B200	768 ± 38	482 ± 31	9	Bone	2230 ± 29	1508 ± 22	12
CB2–30%	1297 ± 22	857 ± 16	5				
CB2–50%	2483 ± 28	1632 ± 16	12				
SB3	3723 ± 25	2448 ± 17	11				

The tissue segmentation, i.e. the process of assigning tissue type and mass density to each voxel, was performed with the SECT image (either the 50 or the 90 kVp scan) and the calibration curve, a two-segment linear relationship (HU-*ρ*), shown on Fig. 2. Different SECT segmentation schemes were derived based on three, four or seven materials to evaluate the effect of the number of media on the segmentation – see Table 3.

**Table 3:** Different segmentation schemes for SECT and DECT for the validation phantom: SECT was segmented with three, four and seven number of materials. For DECT, the segmentation was performed with seven, eight or nine materials. For the ex-vivo mouse, SECT was segmented with three materials and DECT with six

Validation Phantom			
N°	Reference		
9	Air, Adipose, Brain, Spongiosa, Cranium, Cortical Bone		
N°	SECT	N°	DECT
3	Air, Solid Water, Bone	7	Air, Paraffin, Adipose, Breast, Solid Water, Lucite, Bone
4	Air, Adipose, Muscle, Bone	8	Air, Paraffin, Adipose, Breast, Solid Water, Muscle, Lucite, Bone
7	Air, Paraffin, Adipose, Breast, Solid Water, Lucite, Bone	9	Air, Paraffin, Adipose, Breast, Water, Solid Water, Muscle, Lucite, Bone
Ex-vivo Mouse			
3	Air, Brain, Cortical Bone	6	Air, Adipose, Brain, Spongiosa, Cranium, Cortical Bone

### DECT method

**Fig. 4:**
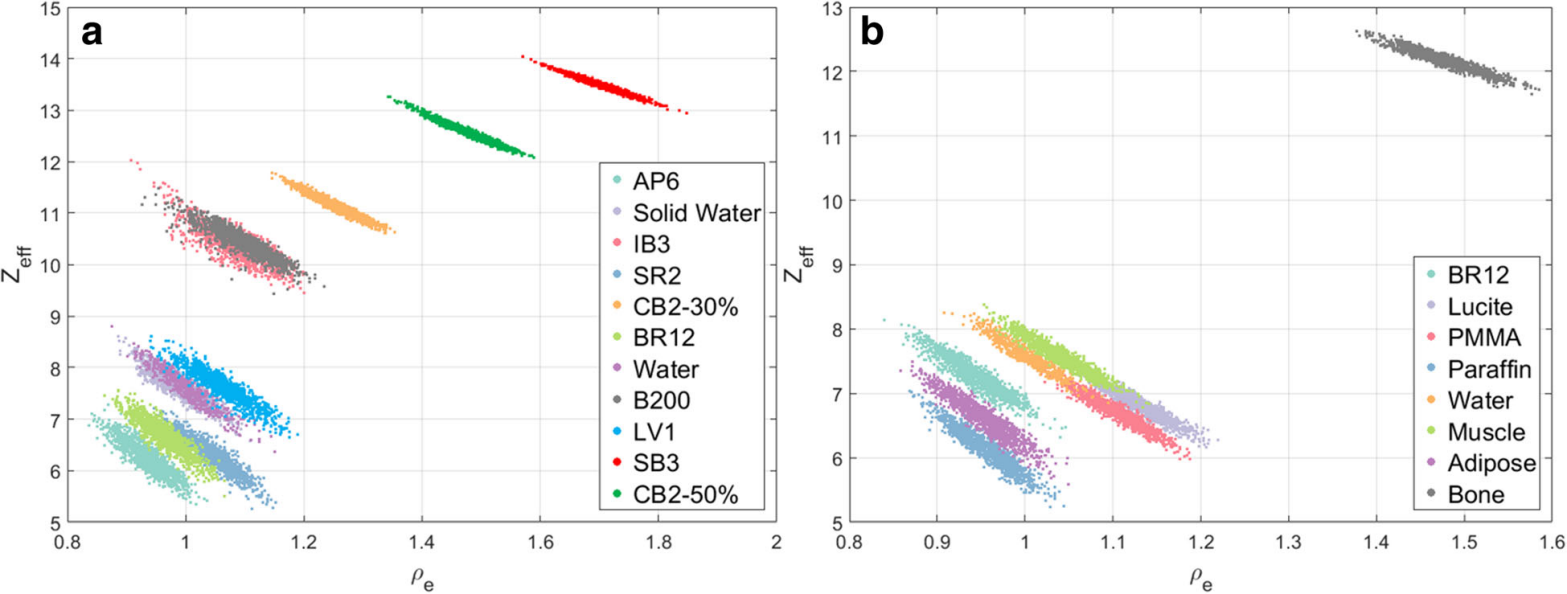
DECT tissue segmentation for all voxels of the (**a**) calibration and (**b**) validation phantoms

For DECT, the CT numbers were extracted from circular regions of interest of the inserts in the four central slices of the high energy (HU_H_) and the low energy (HU_L_) scans. The procedure described by Schyns et al. [11] to determine the *ρ*_*e*_ values, using Saito’s [12] approach, and to extract *Z*_*eff*_, following the method proposed by Landry et al. [13], was adopted. From the HU_L_ and HU_H_ images, *Z*_*eff*_ and *ρ*_*e*_ maps were derived and used for the tissue segmentation. Figure 4 shows the relationship between *Z*_*eff*_ and *ρ*_*e*_ for the materials of the validation phantom. Mass densities were assigned based on the *ρ*_*e*_ images using the (*ρ*, *ρ*_*e*_) relationship (*ρ* = 1.073*ρ*_*e*_ − 0.04, R^2^ ≥ 0.999), the linear relationship between ρ and ρ_e_ was found by fitting the data (least squares method) for the insert materials listed on Table 1. All voxels to which no *Z*_*eff*_ value could be assigned, predominantly located at sharp transitions between air and the solid water bulk, were excluded from the analysis (<0.01% in the regions of interest).

The tissue segmentation was performed with the reconstructed *ρ*_*e*_ and *Z*_*eff*_ of each voxel. The DECT scans at 50 kVp and 90 kVp were used, as this combination has shown to be optimal in terms of *Z*_*eff*_ and *ρ*_*e*_ errors for the X-RAD system with the 3 cm phantoms [11]. The reference values of *Z*_*eff*_ and *ρ*_*e*_, named *Z*_*R*_ and *ρ*_*R*_, and the calculated values from the DECT images, named *Z*_*C*_ and *ρ*_*C*_, were used to assign tissue composition to a voxel. The distance vector between A = [*Z*_*R*_, *ρ*_*R*_] and B = [*Z*_*C*_, *ρ*_*C*_] was calculated and the reference tissue minimizing the distance length was assigned to the voxel. The Mahalanobis distance was used as it is less affected by imaging noise, following the method described by Landry et al. [6].

Different DECT segmentation schemes were also investigated with seven, eight and nine materials. Table 3 shows the schemes.

### Reference phantom

A reference phantom serves as standard for the material segmentation and the dose calculations. It is a mathematical structure created with thresholds and masks for each phantom. It has a single reference value for each material property. The material assignment to the reference phantom is indicated in (Fig. 1a), according to Table 1.

Figure 1d shows the nine materials used, with densities ranging from 0.001 to 1.6 g/cm^3^ (air - bone). In all phantom cases in this study, a broad beam impinges on the phantom from the right-left direction and encompasses its entire volume. The dose is normalized to the maximum dose value in the reference phantom.

All the results are compared to the segmentation and the dose calculation of the reference phantom.

### Ex-vivo mouse specimen

An ex-vivo male mouse was imaged and the same procedures previously described for DECT and SECT, including the calibration phantom parameters, were applied to its CT scans and dose calculations. A region comprising the head of the mouse was selected for this study and material maps with six tissues for DECT and three tissues for SECT were created based on the ICRU Report 44 [14] tissues, listed in Table 4. Using Landry’s method, we chose the closest ICRU tissues to the selected specimen, instead of the materials from the phantom inserts. A fictitious tumour was delineated in a region partially comprising the brain and another organ at risk (OAR), the spinal cord. Table 3 also shows the segmentation schemes for SECT and DECT.

**Table 4:** Tissue data from the ICRU Report 44 [14] for the mouse segmentation

Mouse - ICRU Tissues									
	Material	[g/cm^3^]			Mass percentage (%)				
n°		*ρ*	*ρ*_*e*_	*Z*_*eff*_	H	C	N	O	Z > 8
1	Adipose	0.93	0.951	6.421	11.60	68.10	0.20	19.80	Na (0.1), S (0.1), Cl (0.1)
2	Brain	1.04	1.035	7.578	10.70	14.50	2.20	71.20	Na (0.2), P (0.4), S (0.2), Cl (0.3), K (0.3)
3	Spongiosa	1.18	1.151	10.230	8.50	40.40	2.80	36.70	Na (0.1), Mg (0.2), P (3.4), S (0.2), Cl (0.2), K(0.1), Ca (7.4), Fe (0.1)
4	Cranium	1.61	1.517	12.709	5.00	21.20	4.00	43.50	Na (0.1), Mg (0.2), P (8.1), S (0.3), Ca (17.6)
5	Cortical Bone	1.92	1.780	13.629	3.40	15.50	4.20	43.50	Na (0.1), Mg (0.2), P (10.3), S (0.3), Ca (22.5)
6	Air	0.0012	0.001	7.714			75.47	23.20	Ar (1.28)

The tumour, brain, bone and OAR regions are illustrative structures to investigate possible differences between imaging methods.

### Dose calculations

After the segmentations procedures based on SECT and DECT, dose calculations were performed to verify the impact of these segmentations on the dose accuracy.

The dedicated small animal radiotherapy planning system SmART-Plan (research version 1.5, Precision X-ray, North Branford, CT, United States) was used to calculate the dose distributions [15]. The dose engine used by SmART-Plan is the MC code EGSnrc/DOSXYZnrc [16, 17]. The first step was to provide the material datasets for subsequent use by EGSnrc. Photons were transported down to an energy cutoff (PCUT) of 10 keV and the electron energy cutoff (ECUT) was set to a total energy value of 736 keV (225 kV kinetic energy, meaning no secondary electrons were transported). The photon spectra for the irradiation were calculated using SpekCalc [18, 19] for 100, 160, and 225 kVp, according to the X-ray tube parameters. Exclusively for the ex-vivo mouse simulations, phase-space files for 225 kVp and 100 kVp with a 5 mm beam diameter were used, preserving the above-mentioned characteristics. For the phantom dose calculations, broad beams that covered the phantom were used.

Geometry input files for the phantoms and the animal specimen were created with a Matlab 2016a (The Mathworks, Natick, MA, United States) routine according to the SECT or DECT material segmentation.

The mass density values of liquid and solid water differed only by 2.2%, therefore Solid Water was solely used in both phantoms. For the calibration phantom, material maps were made either using Liver and Inner Bone or Brain and Bone Mineral, and the remaining media, due to the proximity in density values. For the validation phantom, the insert Teflon was not used and Lucite and PMMA were regarded as Lucite, once more due to their similar compositions. Different material maps were also investigated to achieve a better segmentation using fewer media.

The planned dose to water was set to 2 Gy at the isocentre and the number of MC histories with no particle recycling used to achieve a 3% statistical uncertainty for dose calculations with 103.4 × 103.4 × 103.4 μm^3^ voxels was set to 5 · 10^9^ photons for the mini phantoms. The beam field size was set to 3.5 × 1 cm, comprising the selected region of the mini phantom completely. For the mouse, two parallel opposed beams, at 29^o^ and 209^o^, and 9 · 10^7^ particles were used, achieving 1% statistical uncertainty for a dose of 2 Gy at isocentre.

## Results

### SECT segmentation – Number of materials, 225 kVp irradiation spectrum

**Fig. 5:**
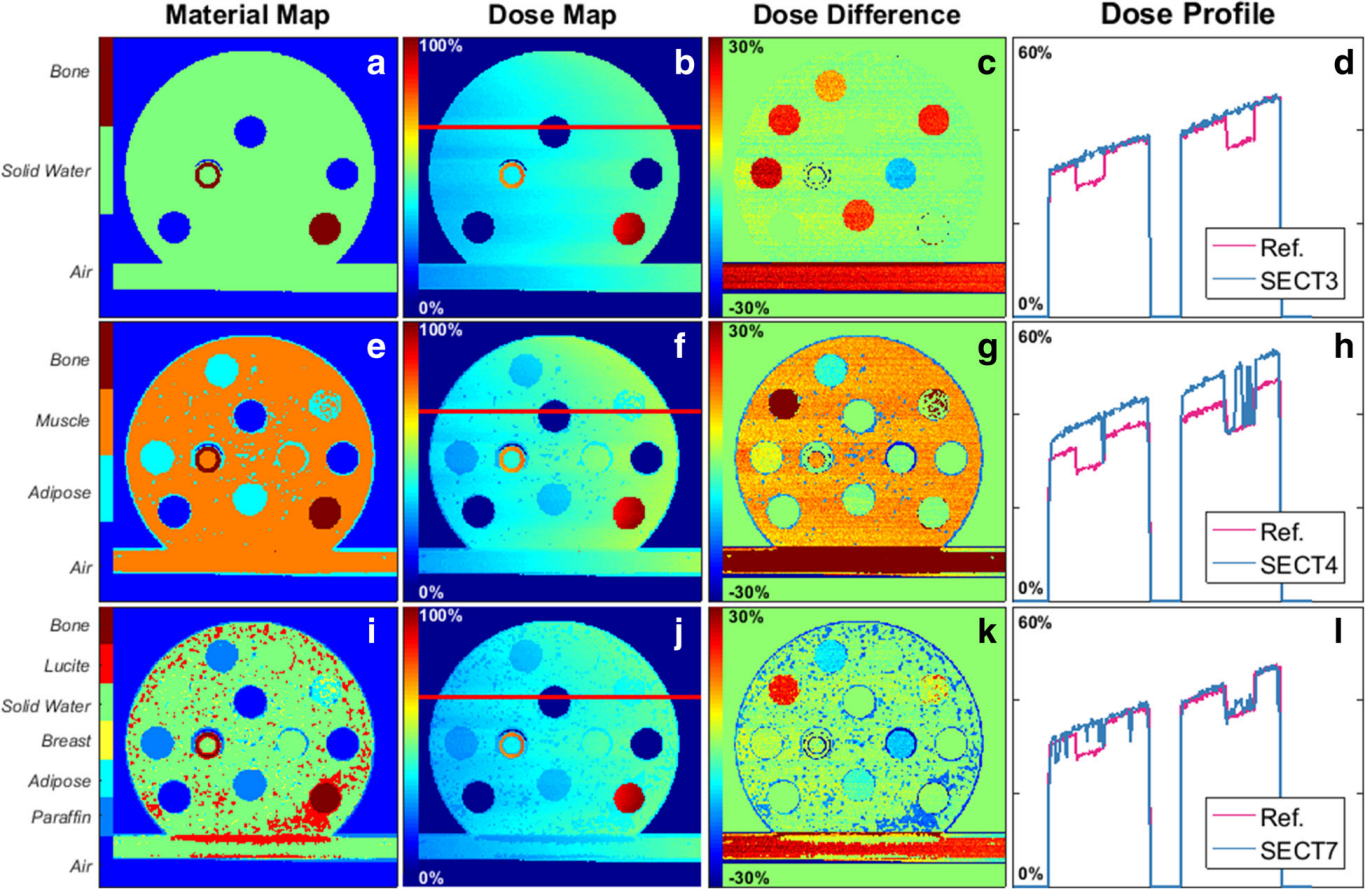
Material maps, dose maps, dose difference, and dose profiles for different SECT segmentation schemes at 50 kVp. **a** Three, (**e**) four, and (**i**) seven different media were used in the three rows. The dose profiles (**d**), (**h**) and (**l**) were obtained from the red line in the images (**b**), (**f**) and (**j**). Images (**c**), (**g**), and (**k**) show the dose difference comparison (ΔD) with the reference, where ∆*D* = [( *D*_*SECT*_ − *D*_*Ref*_)/*D*_*Ref*_] ∙ 100%. The dose maps are normalized to the maximum dose of the reference dose map. The material maps should be compared to the reference phantom, Fig. 1d

Unless stated otherwise, the results presented in this section were generated using the validation phantom. Figure 5 shows the effect of the different numbers of SECT segmentation materials on the MC dose calculations.

The dose to the bulk region of SECT with three materials, SECT3, segmentation agrees with the reference within 1 ± 5% on average. The steps in the profile are due to the Lucite inserts assigned in the Reference phantom but absent in SECT3, their dose differences are 20 ± 1% (Fig. 5c). Figure 7 shows the difference with respect to the reference for all inserts in each SECT scenario.

To increase the efficiency of the dose calculations, no dose was scored in air, thence the regions with zero dose surrounding the phantom and in the air insert.

A different behaviour is shown for the four media segmentation, SECT4, (Fig. 5e-h). Using materials with densities slightly lower (Adipose, 0.967 g/cm^3^) and higher (Muscle, 1.062 g/cm^3^) than Solid Water (1.022 g/cm^3^), the bulk of the phantom is assigned as Muscle, and the inserts Breast, Paraffin and partially the PMMA, are assigned as Adipose. The phantom’s bulk dose differs by 11 ± 7% from the reference and in the inserts, Lucite has the highest difference, 34%, followed by lower differences in the remaining inserts (Fig. 7). This clearly shows that SECT tissue segmentation is highly sensitive to a slight change in the number of materials, and that the selected HU intervals can significantly influence the dose calculations for the kV photon range.

For the seven-material segmentation, SECT7 (Fig. 5i) the misassignment of media has a noise-like appearance in the material and dose maps and profiles (Fig. 5i-l). The material map of SECT7 has 72% of its materials correctly assigned. Regarding the dose, an agreement of 3 ± 5% for the bulk was found and the highest dose difference was once more in Lucite, 21%. It should be stressed that due to the misassignment of media small dose spikes are present throughout the geometry. Assigning a larger number of materials clearly introduces noise in the media assignment and the dose calculations, and the choice of HU intervals also becomes more arbitrary.

For the three cases, Air and Bone are always correctly segmented.

Different material combinations were tested besides the reported ones. The choice for SECT3 and SECT4 was based on the current pre-clinical practice, and SECT7 is shown for further comparison with DECT7. A higher number of SECT materials is not reported as seven fell beyond the limits of the method. The Hounsfield Units histogram, Fig. 3, shows that with a limited number of peaks, a limited number of materials can be assigned using SECT. Another dimension becomes necessary to discern more materials, such as the ρ_e_-Z_eff_ space in DECT.

### DECT method, 225 kVp irradiation spectrum

**Fig. 6:**
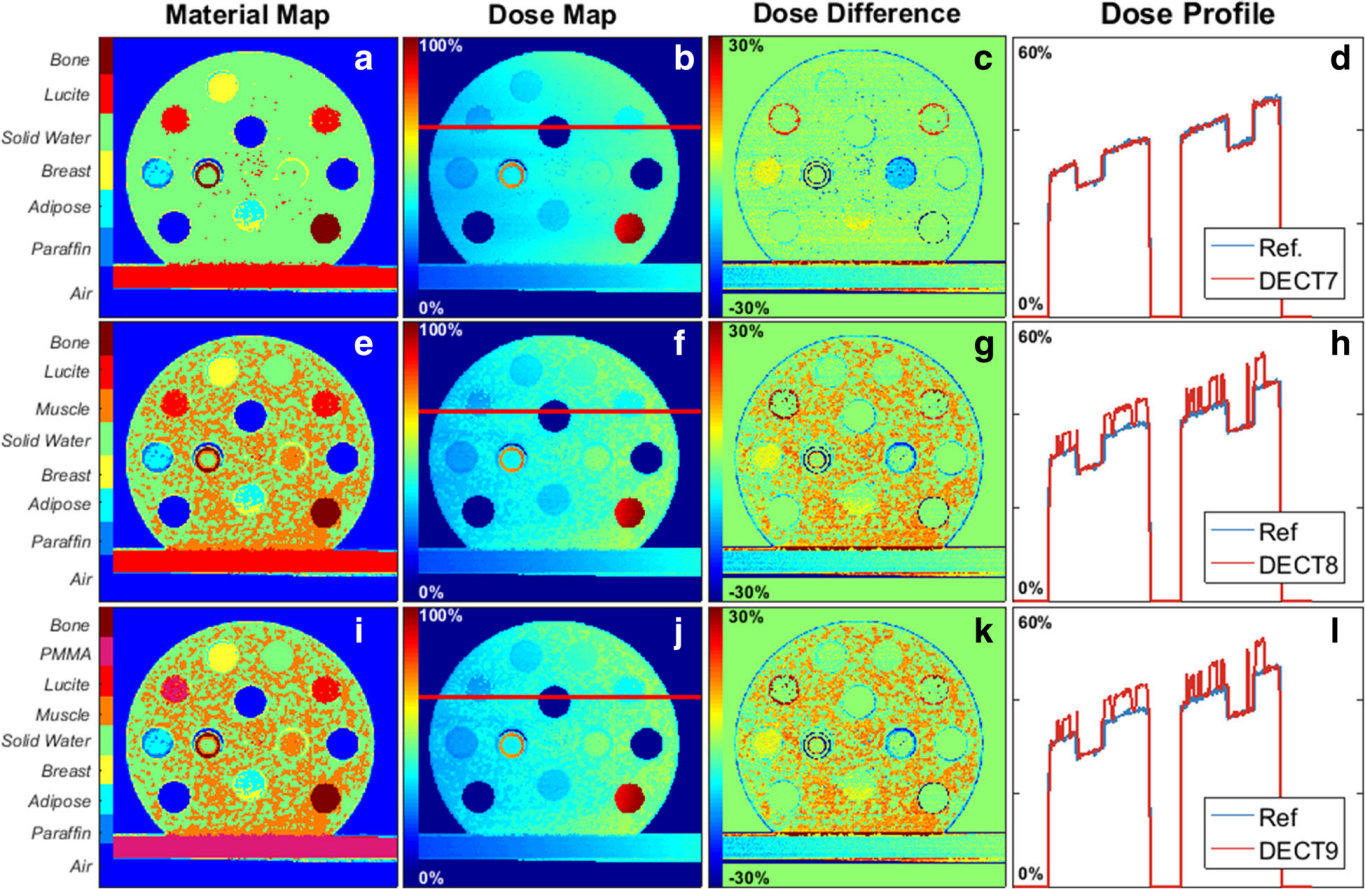
Material maps, dose maps, dose difference and dose profiles for different DECT segmentation schemes. **a** Seven, (**e**) eight and (**i**) nine different media were used in the three rows. The dose profiles (**d**), (**h**) and (**l**) were obtained from the red line in the images (**b**), (**f**) and (**j**). Images (**c**), (**g**), and (**k**) show the dose comparison (ΔD) with reference, where ∆*D* = [ (*D*_*DECT*_ − *D*_*Ref*_)/*D*_*Ref*_] ∙ 100%. The dose maps are normalized to the maximum dose of the reference dose map. The material maps should be compared to the reference phantom, Fig. 1d

For the DECT segmentation, maps with seven (DECT7), eight (DECT8), and nine materials (DECT9) were tested (Fig. 6a, e, i). Similar to SECT, a number of material combinations were tested. The reported DECT combinations were selected based on the highest separation between relative electron density and effective atomic number values, and increased accuracy on the segmentation in comparison to the reference.

**Fig. 7:**
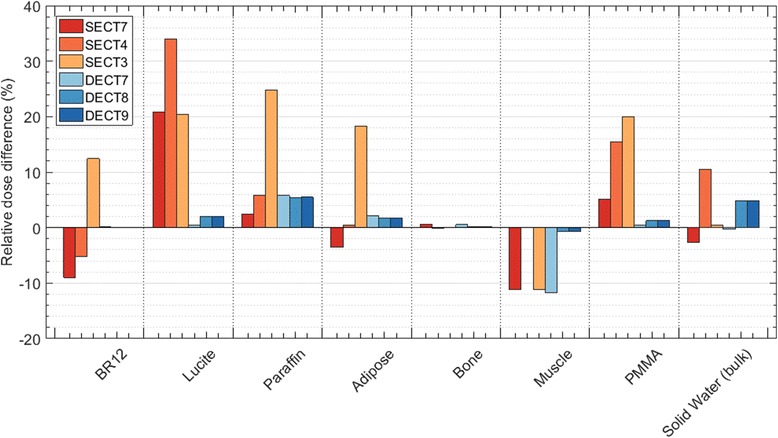
Relative dose difference between the mean doses calculated per insert (and for the bulk of the phantom) of the reference and for SECT and DECT segmentations: SECT3, SECT4, SECT7, DECT7, DECT8, and DECT9. Regions of interest were defined avoiding boundary regions

Increasing the number of materials does not automatically imply a better segmentation for DECT, similar as for SECT. The media misassignment, over 52% for DECT8 and 54% for DECT9, again exhibits noise in the dose maps and profiles (Fig. 6b, f, j, d, h, l) with small dose spikes. The material map of DECT7 was only 16% in disagreement with the reference. For DECT8 and DECT9, the dose difference in the bulk region is, on average, of 5 ± 6% higher than the reference. The insert materials were mostly correctly assigned in the three cases. Figure 7 shows that the highest difference is for the material Muscle in DECT7, 12 ± 1% – Muscle is not one of the media segmented in DECT7. From Fig. 6c, g, k) it is clear that the tissue segmentation scheme may influence the dose accuracy. It should be noted that for DECT the highest dose differences are concentrated in the boundary regions.

Figure 7 shows that dose differences relative to the reference phantom are much higher for the SECT segmentations in comparison to the DECT ones. For kilovolt energies, DECT segmentation yields better results, increasing the dose calculation accuracy when compared to the SECT method.

### Additional irradiation spectra

**Fig. 8:**
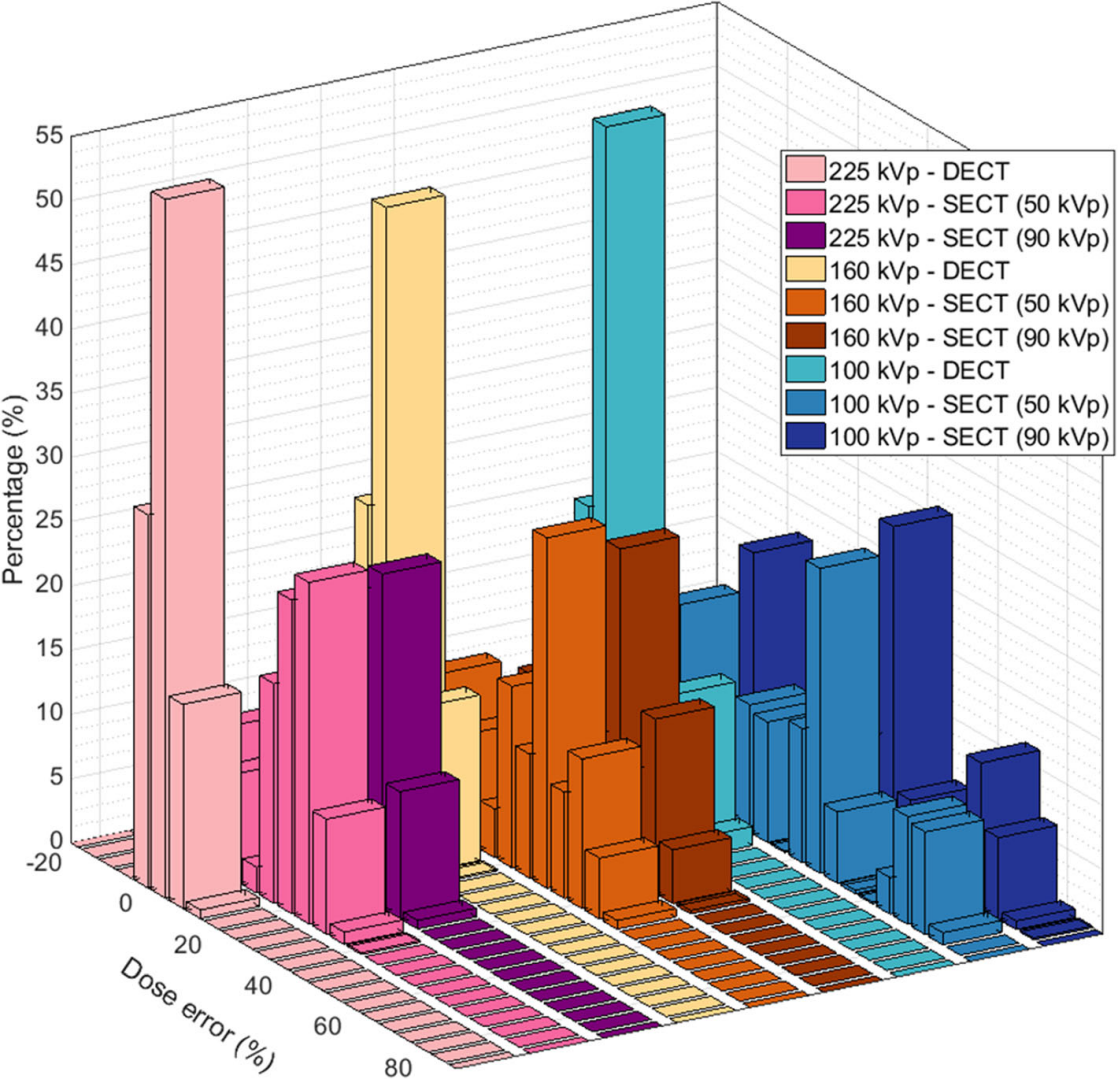
Histogram of the dose disagreement with the reference in the insert regions for DECT, SECT50 and SECT90 at 225, 160 and 100 kVp

In addition to the 225 kVp spectrum, 100 and 160 kVp photon beams were used for the dose calculations. In Fig. 8, a histogram shows the errors on the insert dose values for the SECT and DECT methods of each spectrum. The higher the frequency of events in the zero dose-error bar, the better the segmentation method performed for a specific imaging energy.

Overall, the 225 kVp spectrum presented the best results, followed by the 160 kVp and the 100 kVp. Furthermore, for the three spectra, the DECT method performed better, the zero dose error contained 54, 50 and 53% of the voxels for the 225, 160 and 100 kVp spectra, respectively, and there were no differences higher than 17%, for the 225 and 160 kVp and 27% for the 100 kVp beam. For SECT at 50 kVp, SECT50, the dose differences were as high as 37, 52 and 82%, and at 90 kVp, SECT90, they were as high as 33, 52 and 82%, for the 225, 160 and 100 kVp spectra respectively.

### Ex-vivo mouse

In this section the emphasis is on the difference between the dose calculations based on the two imaging methods as it was not possible to produce a reference ex-vivo mouse – it would require precise knowledge of all its tissues and structures. Although material and dose differences in the bulk of the phantom were shown in the previous section, this concept does not apply to the specimen, as there is no bulk of the mouse.

The SECT (SECT50 and SECT90) and DECT segmentation schemes were used as shown in Table 3. The choice for three media for SECT was based on current pre-clinical practice using 3–4 media [3, 20–24] and the phantom results of Section 2.1. For DECT, six tissues with differences in *ρ*_*e*_ (>11%) and *Z*_*eff*_ (>18%) were chosen as section 2.2 had shown the DECT method to have superior results in the presence of media with a degree of separation in these quantities.

**Fig. 9:**
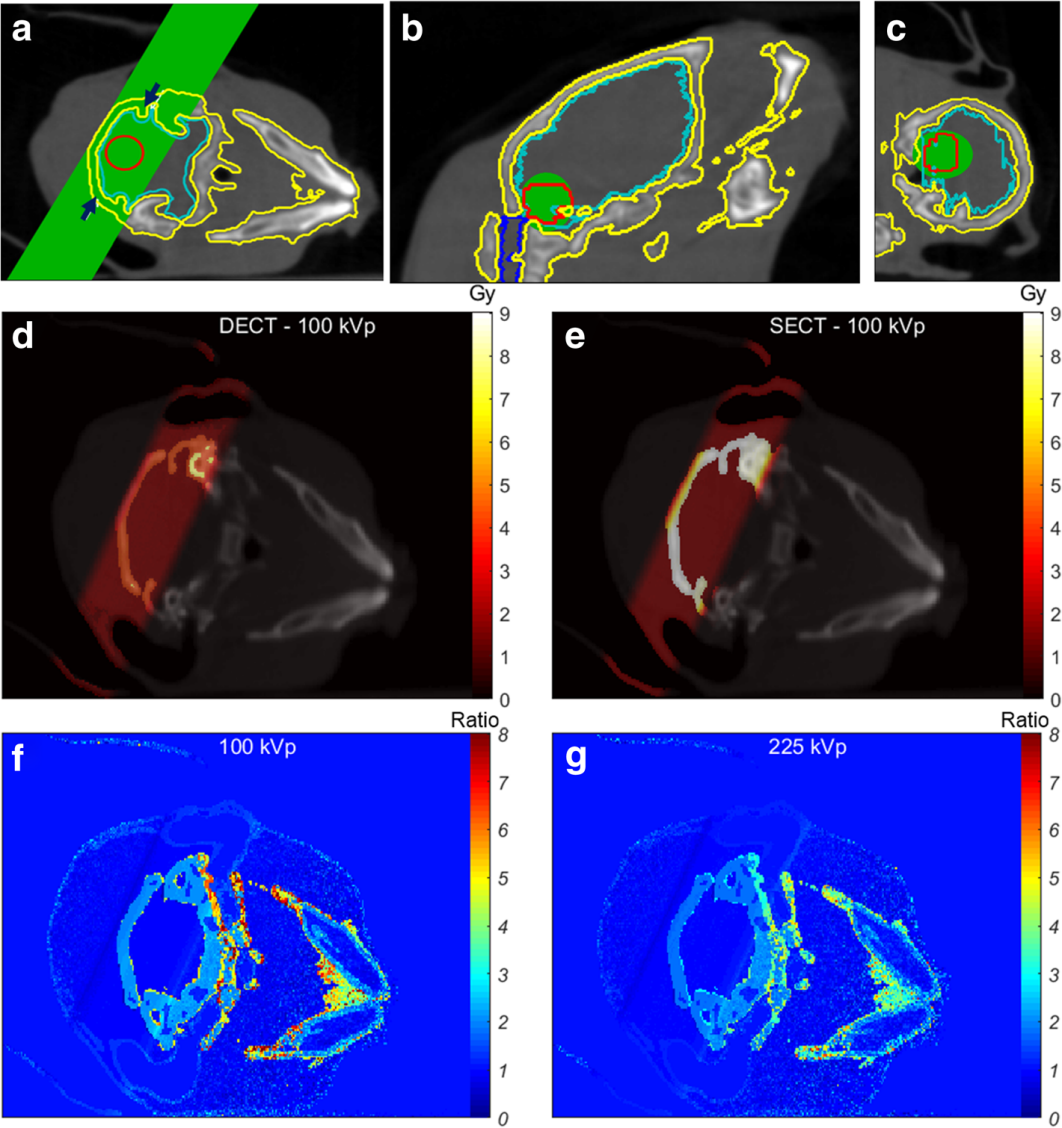
**a** Axial, (**b**) coronal and (**c**) sagittal views of the delineated head of the ex-vivo mouse. The green region in (**a**) and the arrows indicate the beams used for the dose calculations. It encompasses the fictitious tumour (red contour), which is partially in the brain (light blue contour) and the spinal cord (dark blue contour). The elliptic green regions in (**b**-**c**) indicate the target region for the simulation. **d**-**e** show the 100 kVp dose map for DECT and SECT50, and (**f**-**g**) show the ratio between SECT and DECT dose maps for 100 and 225 kVp beams. Due to the similarities between SECT50 and SECT90, only the SECT50 case is shown here

Figure 9a-c shows the axial, coronal and sagittal views of the delineated head of the mouse. The green region in Fig. 9a indicates the position of the parallel-opposed beams. The elliptical green areas in Fig. 9b-c indicate the target volume used for the dose calculations, it encompasses the tumour, which is partially in the brain and the spinal cord. The dose to the target was set to 2 Gy. Fig. 9d-e, shows higher doses for the SECT map, whereas the DECT dose map reveals a gradient due to the presence of different bone media in the same volume. The choice of only one kind of bone implies a high dose for the different media assigned as Cortical Bone in the SECT method. Figure 9f-g shows the dose ratio of SECT and DECT dose maps with accentuated dose differences in Adipose, e.g. close to the outer skin, and in Bone, which are more pronounced for the 100 kVp beam (5.0% of all the voxels in the body contour showed ratios higher than 4).

**Fig. 10:**
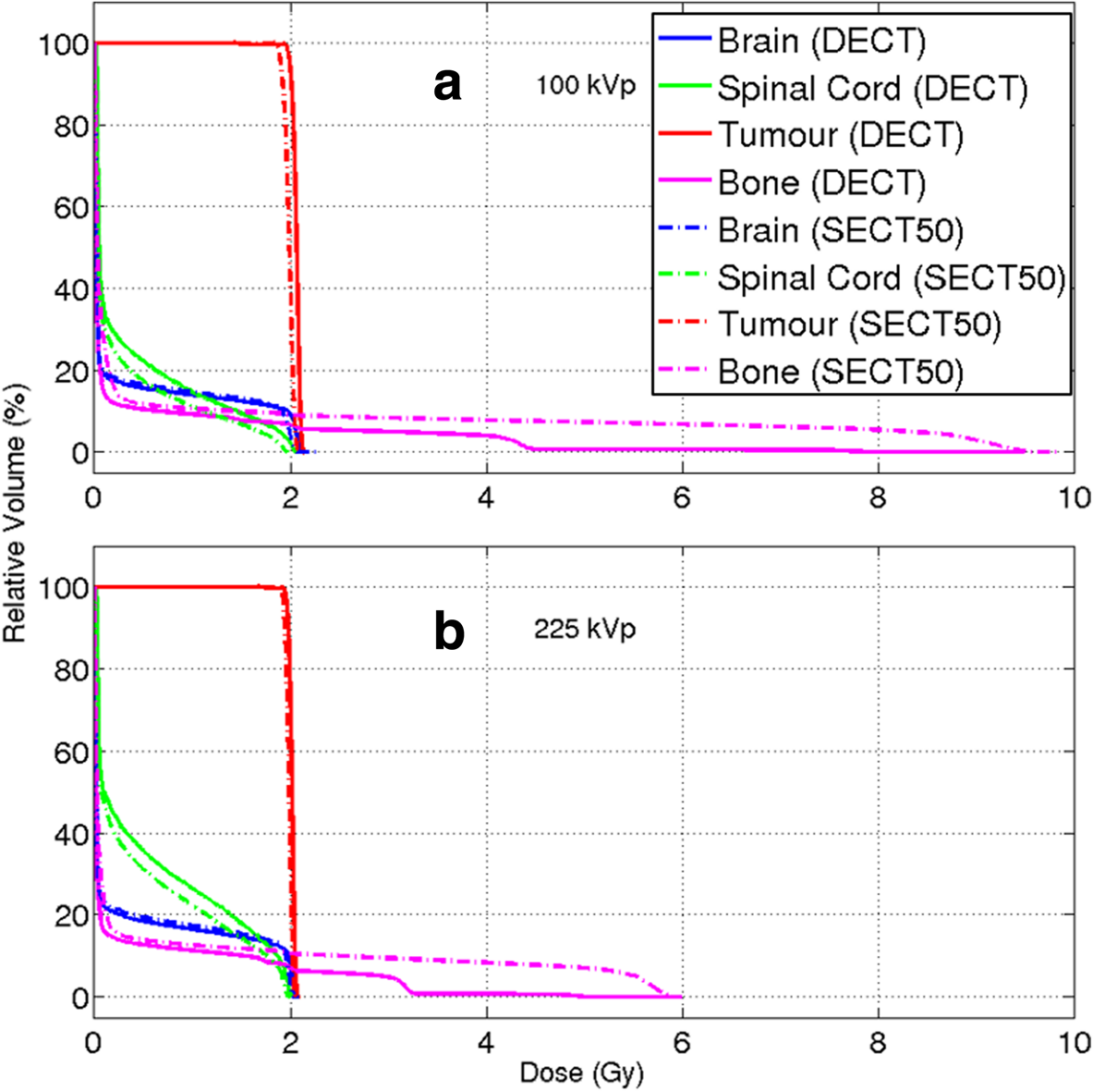
DVHs for the (**a**) 100 and (**b**) 225 kVp beams. Four structures were delineated (as shown in Fig. 9 a-c: Brain, Spinal Cord, Tumour and Bone. The same contours were utilized for all simulations. The solid and the dash-dot lines indicate the DVHs for the DECT and the SECT50 method, respectively

Another way of quantifying the impact of the different segmentations is through Dose Volume Histograms (DVHs). Figure 10a-b shows the DVHs for the 100 and 225 kVp beams. For the bone contour, the dose reaches values three to five times higher than the prescription dose for the 225 and the 100 kVp beams, respectively. The maximum dose was 63% higher for the 100 kVp beam in comparison to the 225 kVp one. For 100 kVp, the presence of higher dose regions is due to a steeper dose gradient required to reach the target value (2 Gy) in the prescription point, for which the same coordinates were specified for the 100 and 225 kVp beams.

Regarding the segmentation method, the SECT curve presents a smooth and steady behaviour as it was segmented with only one type of bone. The DECT curve presents three plateau regions for doses higher than 2 Gy. For 100 kVp, the first region ends with a slope approximately at 4.2 Gy, the second at 7.8 Gy and the last one reaches the maximum dose of 9.5 Gy, and for 225 kVp, the same behaviour is shown at 3.2, 5.0 and 5.9 Gy. It indicates the presence of different bone types used in DECT: Spongiosa, Cranium and Cortical Bone.

The higher energy absorption in bone owing to the exclusive use of the dense Cortical Bone in SECT results in lower doses for the Spinal Cord DVH curves, a structure inside vertebras. Table 5, shows the minimum dose to the hottest 1% (D1), 5% (D5) and 95% (D95) to provide additional information on the uniformity of the dose. The D5 and D1 values for Brain and Tumour are 5% lower for SECT in relation to DECT for both energies. The use of SECT with only one type of bone yielded larger volumes with high doses and the bone choice influenced the dose received by the other structures.

**Table 5:** For each combination of beam energy and imaging method the mean and maximum dose values, the dose values on 95, 5 and 1% of the volume (D95, D5 and D1)

	100 kVp		225 kVp		100 kVp		225 kVp	
	DECT	SECT	DECT	SECT	DECT	SECT	SECT	SECT
Dose (Gy)	Brain				Spinal Cord			
Mean	0.3	0.3	0.3	0.3	0.4	0.3	0.6	0.5
Maximum	2.3	2.3	2.1	2.1	2.0	2.0	2.0	2.0
D95	0.0	0.0	0.0	0.0	0.0	0.0	0.0	0.0
D5	2.1	2.0	2.0	1.9	1.9	1.7	2.0	1.9
D1	2.1	2.0	2.1	2.0	2.0	1.9	2.0	1.9
	Tumour				Bone			
Mean	2.1	2.0	2.0	2.0	0.3	0.7	0.3	0.6
Maximum	2.1	2.1	2.1	2.1	9.5	9.8	5.9	6.0
D95	2.0	1.9	2.0	1.9	0.0	0.0	0.0	0.0
D5	2.1	2.0	2.1	2.0	3.0	8.3	3.0	5.5
D1	2.1	2.0	2.1	2.0	4.4	9.3	3.2	5.8

## Discussion

This study has demonstrated the high impact of incorrect material segmentation on the dose calculation accuracy for kV photon beams employed in small animal irradiators, using the different imaging modalities: SECT and DECT. The effect is aggravated with a decrease in beam energy, due to the increase in the importance of the photo-electric effect with decreasing photon energy, causing materials with different effective atomic numbers to absorb increasingly different fractions of energy in photon beams. For irradiations with photon spectra below 100 kVp, the differences would even be more pronounced.

Although broadly used, there are still certain caveats regarding the SECT method. It is unclear which media should be used for generating the calibration curve and the number of linear segments as well as the position of the tissue boundaries is arbitrary and difficult to establish manually using the HU histogram [3].

DECT showed better overall results in comparison to SECT. The higher number of DECT segmentation media resulted in smaller dose differences in comparison to the reference (Fig. 7) for the phantom cases. Increasing the number of materials in the SECT method yielded more instability, in addition to being a method that has a higher degree of arbitrariness in tissue assignment than DECT. Material boundaries have to be selected based on the distribution of HU, and include a visual inspection of the segmentation result (i.e. in an overlap plot of the CT and the material map), which indicates that inter-individual differences may result. Both modalities have a limit to which adding more materials with similar characteristics ceased providing better segmentation results, and resulted in more noise in the material maps and the dose distributions.

For the mouse case, the choice of Cortical Bone for the SECT method, as is common practice in the literature, resulted in large volumes of tissue receiving high doses. For the DECT method, the choice of more than one kind of bone resulted in lower dose values for the different tissues occupying the same volume, only 1.9% of the bone tissues in DECT were assigned as Cortical Bone (18.5% as Cranium and 79.6% as Spongiosa). For the OAR surrounded by bone in the beam path, the doses were lower when using the SECT method in comparison to DECT, due to the high absorption of the Cortical Bone and the hardening of the beam (low-energy photons were absorbed in the bone), resulting in fewer photoelectric interactions and hence dose deposition in the bone [24]. Therefore, SECT material segmentation may lead to an underestimation of the dose to OAR in the proximity of bone (other examples could be organs in the pelvic area or close to the thoracic spine). In view of these results, with the assumption that bones in small animals might not be as dense or with such elevated atomic number as human bones and considering the interest in studies with lower energies, it can be recommended not to use Cortical Bone when performing SECT segmentation. The choice of Spongiosa would be more appropriate and additional bone types may need to be considered for specific regions, as mouse bones are very flexible, in composition possibly closer to human cartilage, which has less phosphorus and calcium than Cortical Bone. For studies with lower energies, the choice becomes more important if higher doses to bony structures are not intended. It is also beneficial to employ harder beam filters.

DECT with three or four tissues is not reported. The method’s advantage lies in the possibility of exploring different segmentations based on higher number of tissues. A reduced number of materials would not benefit this site.

In the soft tissue range, the benefits of DECT for the energy 225 kVp are relatively small. For small animals such as mice, the affected regions lie mainly in bony structures. For larger animals, cumulative errors could have a larger role and need further considerations. Improvements in tissue segmentation from DECT are needed for lower photon energies and proton beams in all tissues.

A source of uncertainty in this study is the presence of noise in the CT scans. In Fig. 1b-c, artefacts can be seen in the bone insert, and the bulk of the phantom seems to have a texture instead of consisting of a uniform medium. The CT values of the entire region are irregular, 42 ± 62 and 16 ± 57 HU for 50 and 90 kVp scans. For DECT, the *Z*_*eff*_ image is the most affected, with a noisy appearance and the bulk medium with a mean *Z*_*eff*_ value of 8.0 ± 0.4 (ranging from 6.0 to 10.7), which encompasses many of the soft tissues used in the segmentation and makes it especially hard to distinguish between Water, Solid Water and Muscle, which also have densities close together. The large misassignment of materials on DECT8 and DECT9, using materials with similar characteristics (*Z*_*eff*_ and *ρ*_*e*_) can be partially attributed to image noise. The image noise and misassignment follow a similar pattern on Fig. 6f and j. The CT projections were reconstructed with a simple FDK backprojection algorithm. The usage of an iterative reconstruction algorithm with beam hardening and artefact correction kernels could improve the effect of noise on the images and provide superior material segmentation when performing DECT [25].

The boundary regions of the phantom and the inserts presented the highest source of errors for DECT. This can be explained as a partial volume effect: as two contiguous materials partially fill a voxel, they are combined into voxels that do not correspond to the CT numbers of either of the materials. This will play a larger effect in phantoms with small air gaps than in animals. Another possible and complementary explanation is that the images should have a perfect overlap with the reference phantom, a small misregistration would provide substantial differences. This is a feature DECT is sensitive to, while it plays no role for SECT images. For small shifts between two scans, due to setup or animal movement, rigid image registration could be used if potential HU errors from interpolations are minimal.

Dose calculations in human radiotherapy in the megavoltage photon energy range are not very sensitive to tissue compositions, however, in the kV range used in brachytherapy [6] and in preclinical studies mimicking human radiotherapy at the level of rodents it becomes a potential cause of uncertainties [21]. A final issue that deserves attention is that in the present study and, in general, the small animal radiobiology literature, specimens are segmented with human-like tissues. It is reasonable to assume that either knowing the actual composition or deriving a relationship between human and animal tissues should benefit the dose calculation accuracy and the absorbed dose for the photon energies used in this study.

## Conclusions

The feasibility of dual-energy CBCT imaging for kV dose calculations in pre-clinical studies was presented. Images were obtained using well-separated X-ray spectra were acquired with an on-board imager and different segmentation schemes were tested. The DECT method enabled the employment of a higher number of materials increasing accuracy in dose calculations. In phantom studies, both SECT and DECT presented a limit to which adding materials resulted in more imaging noise in the material maps and the dose distributions. SECT performed best with three materials and DECT with seven for the phantom case. With lower beam energies, the effect of incorrect segmentation on the dose calculations was worse, due to the importance of the photoelectric effect for the kV energy range. DECT segmentation offers the distinct advantage of taking into consideration the effective atomic number of the media. For the ex-vivo specimen, the dose calculations derived from the SECT method showed larger volumes with high doses. For kV energies, the use of DECT segmentation combined with the choice of a bone with low density and atomic number is recommended.

## Abbreviations

CBCT: Cone beam computed tomography
CT: Computed tomography
DECT: Dual energy computed tomography
DECT7, DECT8, DECT9: Dual energy computed tomography segmented with 7,8 or 9 materials
DHV: Dose volume histogram
FDK: Feldkamp-Davis-Kress
HU: Hounsfield units
HU_H_: High energy scan
HU_L_: Low energy scan
kV: kilovoltage
MC: Monte Carlo
MV: Megavoltage
OAR: Organs at risk
RT: Radiotherapy
SECT: Single energy computed tomography
SECT3, SECT4, SECT7: Single energy computed tomography segmented with 3,4 or 7 materials
SECT50, SECT90: Single energy computed tomography imaged with 50 or 90 kVp
Z_eff_: Effective atomic number
ρ: Mass density
ρ_e_: Relative electron density

## Footnotes

1
*ρ*_*e*_ = (*N*_*A*_ *ρZ*/*A*)/(*N*_*A*_ *ρ*_*w*_ *Z*_*w*_/*A*_*w*_), where *N*_*A*_ is the Avogadro’s number, *ρ*, *Z* and *A* are the mass density, atomic number, and atomic mass of a material, while the subscript *w* indicates water

2
${Z}_{eff}={\left({\Sigma}_i{w}_i{Z}_i^{\beta}\right)}^{1/\beta }$, where *w*_*i*_ is the weight fraction of element *i* with atomic number *Z*_*i*_ and *β* = 3.31 [6]
